# Achieving High‐Precision Attenuation Coefficient Measurement in Optical Coherence Tomography

**DOI:** 10.1002/jbio.202400395

**Published:** 2025-01-16

**Authors:** Linda B. Neubrand, Xavier Attendu, Ton G. van Leeuwen

**Affiliations:** ^1^ Department of Biomedical Engineering and Physics Amsterdam UMC, Location University of Amsterdam Amsterdam The Netherlands; ^2^ Atherosclerosis and Ischemic Syndromes Amsterdam Cardiovascular Sciences Amsterdam The Netherlands; ^3^ Imaging and Biomarkers Cancer Center Amsterdam Amsterdam The Netherlands; ^4^ Department of Engineering Physics Polytechnique Montreal, Centre d'Optique Photonique et Lasers Montreal Quebec Canada

**Keywords:** attenuation coefficient, Cramér–Rao lower bound, curve‐fitting, depth‐resolved estimation, Fisher matrix, optical coherence tomography, precision

## Abstract

In this study, we aim to validate the analytical Cramer‐Rao lower bound (CRLB) equation for determining attenuation coefficients using a 1310 nm Optical Coherence Tomography (OCT) system. Our experimental results successfully confirm the validity of the equation, achieving unprecedented precision with a standard deviation below 0.01 mm^−1^ for intralipid samples. Furthermore, we introduce a systematic framework for attaining high precision in OCT attenuation measurements.

## Graphical abstract

We aim to determine the attenuation coefficients using a 1310 nm Optical Coherence Tomography (OCT) system with an unprecedented standard deviation below 0.01 $mm^{-1}$ for intralipid samples. Our experimental results validate the analytical Cramer‐Rao lower bound equation. Additionally, we present a systematic framework for achieving high precision in OCT measurements.

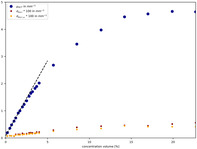

## 1 Introduction

Optical Coherence Tomography (OCT) is an imaging technique that utilizes low‐coherence interferometry to provide detailed cross‐sectional views of samples. Widely applied in medical diagnostics but also in other fields like art conservation, OCT yields high‐resolution depth‐resolved images with axial resolutions typically in the range of 5 to 15 μm.

Beyond visualization of the organization and structure of the tissue, OCT signals also contain information on physiological properties, for example, blood content and blood flow [1, 2]. Analysis of the OCT signals can also provide the optical properties of the tissue, for example, the attenuation coefficient ($\mu_{\mathrm{OCT}}$), the ballistic light intensity decay due to absorption ($\mu_{a}$) and scattering ($\mu_{s}$) within the sample [3, 4]. These coefficients are valuable for tissue characterization and cancer detection [2, 5, 6], particularly in disciplines such as cardiology, dermatology, and urology [7, 8, 9, 10, 11, 12, 13, 14].

A precise method to determine the attenuation coefficient is an important boundary condition to extract reliable optical information from tissues and to use that information to distinguish tissue type or to determine physiological information.

To deepen our understanding of the precision in measuring OCT‐attenuation, we recently introduced a theoretical framework [15]. In this framework, we utilized a Maximum Likelihood approach to derive the Cramer‐Rao lower bound (CRLB) for the standard deviation of OCT‐attenuation (${\sigma_{\mu }}_{\mathrm{OCT}}$). Additionally, we formulated an analytical expression defining the minimum precision required for determining the attenuation coefficient through curve fitting (CF), which is a conventional method for extracting the attenuation coefficient using an appropriate signal model [15]. This model not only encompasses the optical properties of interest but also integrates parameters that characterize the OCT system itself, introduced by the confocal point spread function.

In our previous work, we validated our theoretical framework and analytical equation using simulated OCT data. In this manuscript, we aim to experimentally confirm the validity of the theoretical framework and analytical equation, providing a proof of concept in a homogeneous medium. To achieve this, we investigated the OCT‐attenuation across a range of intralipid volume concentrations, ranging from 0.2% to 22.7%. Utilizing a self‐built OCT system operating at a central wavelength of 1310 nm, we determined the attenuation coefficients of intralipid volume concentrations with an unprecedented precision of 0.01 mm^−1^. Additionally, we successfully validated our previously proposed analytical equation of the expected precision and the associated theoretical framework.

## 2 Theory

When investigating intralipid under the assumption of a single scattering approximation, the OCT averaged mean intensity signal $\left\langle I\right\rangle$ versus optical depth z is best described as a single exponential decay [16]:

(1) $$\left\langle I\left(z,\alpha ,z_{f},z_{R},\mu_{\mathrm{OCT}},\zeta \right)\right\rangle =\alpha \cdot T\left(z-z_{f},z_{R}\right)\cdot H\left(z\right)\cdot \exp\left(-2\frac{\mu_{\mathrm{OCT}}}{n}z\right)+c$$

This equation includes the sensitivity roll‐off in depth $H\left(z\right)$ and the confocal point spread function [17].

(2) $$T\left(z-z_{f},z_{R}\right)=\frac{1}{{\left(\frac{z-n^{2}z_{f}}{n^{2}z_{R}}\right)}^{2}+1}$$

The latter function is dependent on the relative distance to the focal point position $z_{f}$ and the Rayleigh length $z_{R}$, both measured in air, and n the refractive index of the tissue. The conversion factor $\alpha =\eta \cdot \mu_{b,\mathrm{NA}}$ includes the detector response (η) and the backscattering coefficient ($\mu_{b,\mathrm{NA}}$) within the numerical aperture (NA). c = $\left\langle c\right\rangle$ is the mean noise floor, which we assume being independent of depth. Typically, the offset c is determined by averaging a noise area at the end of the imaging range. Subtracting this offset yields an equation dependent on four parameters to $\theta =\left[\alpha ,\mu_{\mathrm{OCT}},z_{f},z_{R}\right]$.

Moreover, the sensitivity roll‐off $H\left(z\right)$ can be calibrated out and the focal parameters $z_{f}$ and $z_{R}$ can be pre‐determined, for example, by mirror measurements at different depths, a beam profiler or directly from diluted intralipid samples [2]. The parameters of interests are then reduced to $\theta_{\text{corr}}=\left[\alpha ,\mu_{\mathrm{OCT}}\right]$ and Equation (1) simplifies to

(3) $$\left\langle I\left(z,\alpha ,\mu_{\mathrm{OCT}}\right)\right\rangle =\alpha \cdot \exp\left(-2\frac{\mu_{\mathrm{OCT}}}{n}z\right)$$

One common approach to extract the attenuation coefficient and its standard deviation is to select an axial fitting range (AFR) and apply a nonlinear least squares curve fit (NLLS) using Equation (1) or (3). To determine the highest achievable precision of $\mu_{\mathrm{OCT}}$ in our measurements, we calculate the Fisher Information Matrix ($F_{\theta }$) [15, 18, 19]:

(4) $${\left(F_{\theta }\right)}_{\mathrm{kl}}=\sum_{i=1}^{M}\frac{N}{\sigma_{I}^{2}\left(z_{i}\right)}\left(\frac{\partial \left\langle I\left(z_{i}\right)\right\rangle }{\partial \theta_{k}}\frac{\partial \left\langle I\left(z_{i}\right)\right\rangle }{\partial \theta_{l}}\right){|}_{\theta =\theta_{0}}$$

Here, *M* represents the number of independent sampling points, *N* is the number of A‐scans used for averaging and $\sigma_{I}^{2}\left(z_{i}\right)$ is the variance of intensity at each depth point $z_{i}$. The partial derivatives $\left(\frac{\partial \left\langle I\left(z_{i}\right)\right\rangle }{\partial \theta_{k}}\;\text{and}\;\partial \frac{\left\langle I\left(z_{i}\right)\right\rangle }{\partial \theta_{l}}\right)$ represent the sensitivity of the intensity to changes in the parameters $\theta_{k}$ and $\theta_{l}$, respectively, evaluated at a reference point $\theta_{0}$, which are the true values of the parameters of interest. Please note that we make the assumption that our signal in Equation (1) conforms to a normal distribution. Typically, single intensity signals exhibit an exponential distribution. However, achieving a normal distribution is feasible by averaging laterally more than 30 independent A‐scans [15, 20].

Upon obtaining the Fisher matrix, we can determine the CRLB for the parameters of interest θ by the square rooting the diagonal elements of the inverse Fisher matrix:

(5) $$\text{CRLB}=\sqrt{\mathrm{diag}(F_{\theta }^{-1}})$$

This CRLB signifies the highest precision achievable based on the given model or equation. For further insights into the CRLB calculation, we have provided detailed analyzes in [15, 19]. The analytical solution for the CRLB of the standard deviation of the OCT‐attenuation extracted from a 2 parameter fit (Equation 3) is given by [15].

(6) $$\sigma_{\mu_{\mathrm{OCT}},\text{an}}=\sqrt{F_{\theta }^{-1}}=\frac{1}{|\mathrm{AFR}|}\sqrt{\frac{3}{M\cdot N}}$$

that provides a rigorous, fast and easy‐to‐use measure for signals with an SNR above 20 dB [4].

The analytical CRLB is inversely proportional to the square root of the number of pre‐averaged A‐scans (*N*), the square root of the number of independent sample points used for signal acquisition (*M*), and the length of the axial fitting range (∣AFR∣).

In addition to precision, accuracy is also an important factor. Aernouts et al. [21] proposed a formula to estimate the value of the scattering coefficient for various intralipid concentrations, taking into account the effect of concentration dependent scattering:

(7) $$\mu_{s,\text{theo}}=\frac{f_{v}\cdot \mu_{s,\text{indep}}\left(\lambda \right)}{0.227}\cdot \frac{{\left(1-f_{v}\right)}^{p\left(\lambda \right)+1}}{1+f_{v}\cdot {\left(p\left(\lambda \right)-1\right)}^{p\left(\lambda \right)-1}}$$

Where the first term describes the linear relation between the independent scattering coefficient $\mu_{s,\text{indep}}=b\cdot 10^{9}\cdot {\left(\lambda \right)}^{-2.59}$, $b=1.868\;mm^{-1}$, and the volume fraction $f_{v}$ as a function of wavelength $\lambda \;\left[\mathrm{nm}\right]$. The second term describes the concentration dependent scattering and includes the effect of the small distances between the scattering particles via the packaging factor $p\left(\lambda \right)=1.31+a\cdot \lambda$, $a=0.0005481\;nm^{-1}$.

## 3 Methods

We conducted a study to investigate the OCT‐attenuation across various concentrations of intralipid volume (intralipid 20, Fresenius Kabi Nederland BV). Dilutions resulted in particle volume concentrations ranging from 0.01% to 22.7%, covering a wide concentration spectrum. These concentrations (0.01%, 0.025%, 0.05%, 0.2%, 0.5%, 0.7%, 1.0%, 1.2%, 1.5%, 1.7%, 2.0%, 2.2%, 2.5%, 2.8%, 3.0%, 3.2%, 3.5%, 3.7%, 4.0%, 5.7%, 8.5%, 11.4%, 14.2%, 17.0%, 19.9%, and 22.7%) were generated by mixing intralipid with milliQ water.

### 3.1 Instrumentation

Intralipid measurements were performed using a custom‐built OCT system operating at a central wavelength of 1310 nm with a bandwidth of 100 nm. The system features a pixel step size of 9.4 μm and an axial resolution of 15.2 μm, determined from the full width at half maximum (FWHM) of the intensity of a mirror measurement. Prior to measurements, samples underwent vortexing to prevent fat particle clustering. For the measurements on the intralipid samples, care was taken to maintain a consistent pixel depth of the surface in order to prevent any shifts in confocal parameters between the different measurements (concentrations). We further used an achromatic sample arm lens (Thorlabs AC254‐045‐C) with a focal length of 45 mm. Data processing and analysis were carried out using Python 3.11.

### 3.2 Method Overview

**FIGURE 1:**
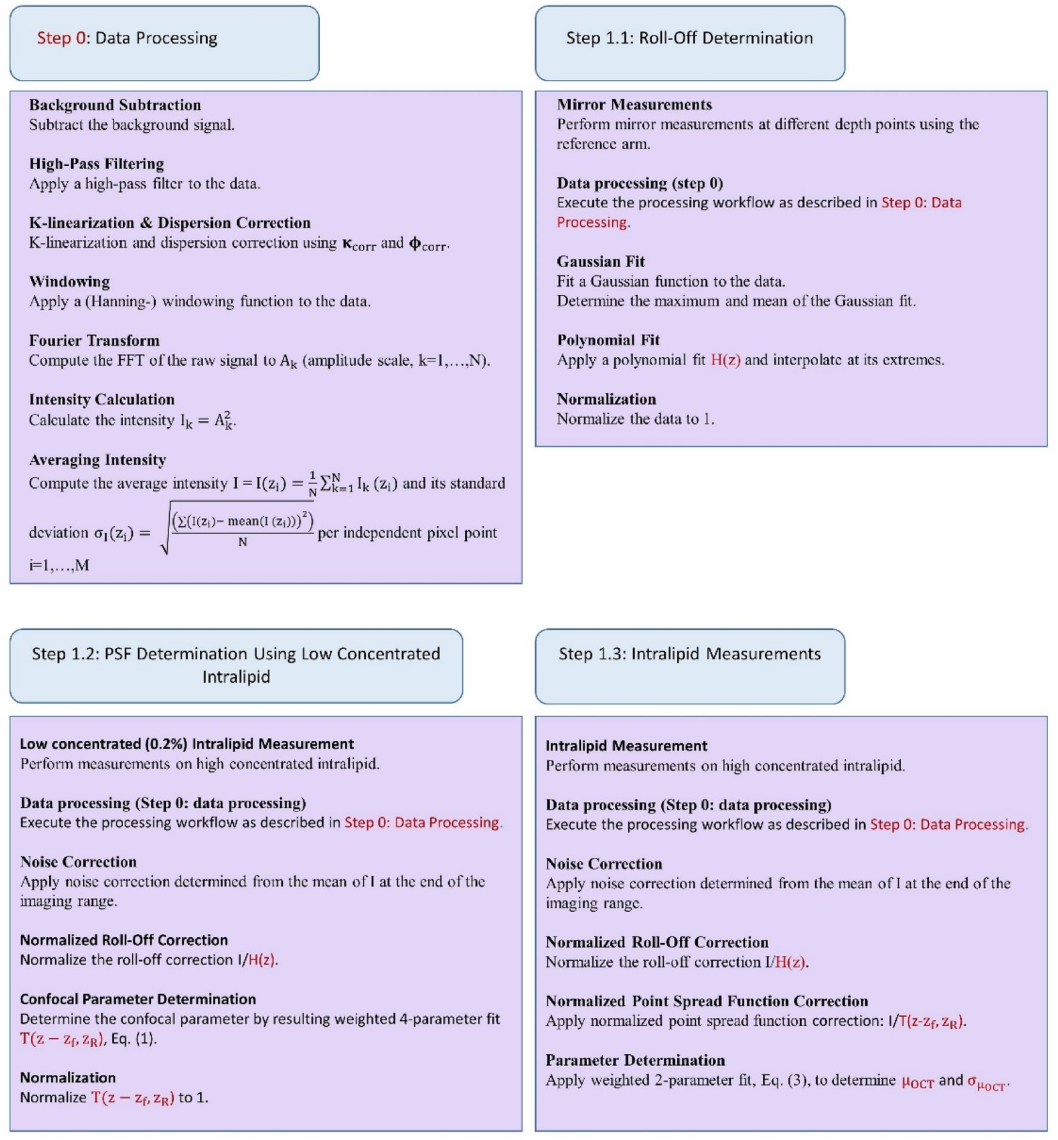
Method 1—Sequential outline of data processing and analysis.

**FIGURE 2:**
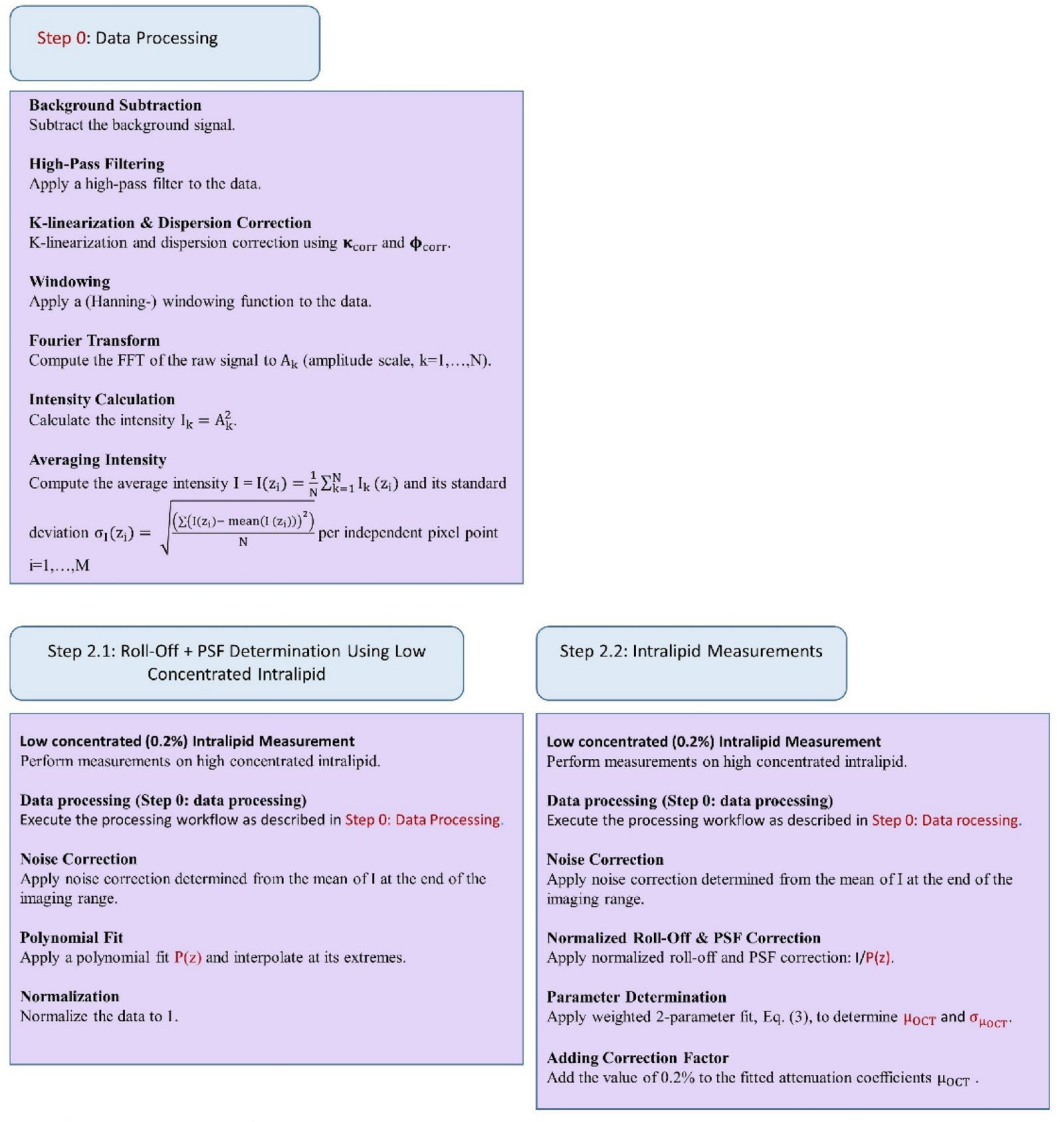
Method 2—Sequential outline of data processing and analysis.

Two methods, illustrated in Figures 1 and 2, were employed to process and analyze the OCT data acquired from the intralipid samples. The primary distinction between these methods lies in the steps involved in correcting for roll‐off and point spread function (PSF) effects. Both methods share an identical initial step, Step 0, to determine a k‐linearization and dispersion vector, which are subsequently used to k‐linearize the data and correct for dispersion.

#### 3.2.1 Step 0: Data Processing

Background measurements recorded without a sample (i.e., in air) were subtracted from the raw data. Next, FIR high‐pass filtering was applied to the signal using Python's Firwin function. Following this, dispersion correction and k‐linearization were applied as described in [22]. Hanning windowing preceded the Fast Fourier Transform (FFT) process, and the resulting individual A‐scans (amplitude as function of depth) were squared for intensity scale conversion. To minimize correlation between adjacent A‐scans, every 20th A‐scan was averaged, resulting in one meanT A‐scan (and one standard deviation per pixel point) per intralipid concentration. A total of 12 500 independent A‐scans were used for averaging for most analysis, unless indicated otherwise.

### 3.3 Method 1

#### 3.3.1 Step 1.1: Roll‐Off Determination

Roll‐off measurements were performed by placing the sample mirror in focus and changing the length of the reference arm to scan the peak across the entire imaging range.

Next, data processing, Step 0, was applied and the mean noise floor was estimated at the imaging end range and subtracted from the processed data. Finally, Gaussian fitting yielded peak heights, which, in turn, were fitted by a 10‐order polynomial, interpolated, and normalized to one.

#### 3.3.2 Step 1.2: PSF Determination Using Low Concentrated Intralipid

First, we used samples with a low concentration intralipid particles (0.01%, 0.025%, 0.05%, 0.2%, and 0.5%) to determine the confocal parameters (using the 4‐parameter model, Equation 1).

Data was processed as accordingly described in Step 0. Confocal parameters $z_{R}$ and $z_{f}$ were extracted by fitting the 4‐parameter model, Equation (1), to the mean (*N* = 12 500), noise corrected A‐scan obtained from the processed data of the 5 intralipid concentrations. Based on the precision of the confocal parameters (using the Cramer‐Rao analysis, see results), we chose to use the confocal parameters of the OCT measurements of the 0.2% Intralipid. The resulting point spread function, Equation (2), was normalized to one.

#### 3.3.3 Step 1.3: Intralipid Measurements

Next, we performed measurements of the intralipid samples with particle concentrations ranging from 0.2% to 22.7%, again while taking care that the intralipid surface was at the same pixel depth for all measurements. The processing steps were the same as those outlined in step 0.

The resulting variance (standard deviation squared), $\sigma_{I}^{2}$, per pixel point was later utilized in the numerical CRLB calculation for the confocal parameters.

After noise subtraction, the intralipid data were divided by the roll‐off and point spread function. Finally, a two‐parameter fit (Equation 3) was applied to extract the backscattering amplitude (α) and attenuation coefficient ($\mu_{\mathrm{OCT}}$) values. The covariance matrix, also retrieved from the fit, was used to determine their respective standard deviations by taking the square root of its diagonal elements.

### 3.4 Method 2

Step 0 and Step 2.2 follow a methodology analogous to Method 1 and are therefore not reiterated here. The main deviation occurs in Step 2.1.

#### 3.4.1 Step 2.1: Roll‐Off & PSF Determination Using Low Concentrated Intralipid

Given the low scattering concentration of the 0.2% intralipid solution, the processed averaged (*N* = 12 500) A‐scan primarily contains information on the roll‐off, the point spread function (PSF), and the absorption coefficient. Consequently, instead of determining the roll‐off and PSF individually, a normalized polynomial fit of this mean A‐scan served as calibration reference in Step 3 for processing the intralipid data.

To facilitate a direct comparison of attenuation values obtained from both methods, we added the attenuation value of 0.19 $mm^{-1}$ extracted for the 0.2% intralipid concentration ($\mu_{\mathrm{OCT},0.2}$) from Method 1 as an offset to the results obtained from Method 2.

### 3.5 Cramér‐Rao Lower Bound Calculations

We conducted a numerical calculation of the 4‐parameter CRLB to investigate the relationship between the CRLB of the standard deviation of the confocal parameters and the intralipid concentration‐dependent attenuation coefficient.

We utilized the CRLB to determine the optimal intralipid concentration data for the PSF in step 1.2 of Method 1, as well as for calibration intralipid concentration used in step 2.1 of Method 2. In this calculation, we considered the outcomes for $\mu_{\mathrm{OCT}}$, $z_{R}$, and $z_{f}$ from the 4‐parameter fit, Equation (1) (*c* = 0), as the “true” (ground truth) parameter values $\theta_{0}$ in Equation (3). These values were utilized as input in our calculation. We further utilized the variance (square of the standard deviation) per pixel point of each averaged concentration. To ensure accuracy, we corrected its offset (*c* = 0, Equation 1) by subtracting the mean noise derived from the data of the highest intralipid concentration from the individual standard deviation curves.

Furthermore, we compared the standard deviation of the attenuation coefficient extracted from the 2‐parameter fit of the intralipid data with the analytical equation described in Equation (6), utilizing *N* = 25 to *N* = 12 500 and *M* ranging from 129 to 600 pixels (depending on the intralipid concentration).

## 4 Results

**FIGURE 3:**
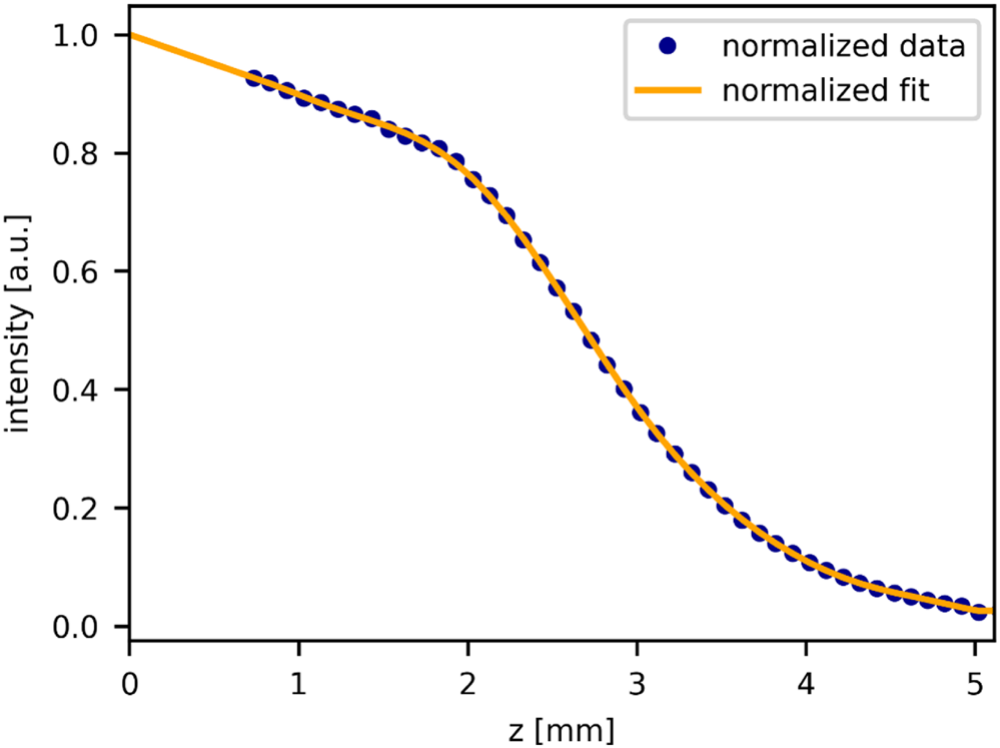
Roll‐off data (blue dots) and its 10th‐order polynomial fit (orange line), normalized to 1. The plot demonstrates a gradual decrease in intensity up to 1.9 mm, followed by a sharper decline thereafter.

In Figure 3, the roll‐off data is presented alongside its corresponding 10th‐order polynomial fit, both of which have been normalized to 1 for clarity of comparison. The graph visually depicts a gradual decrease in the total roll‐off decay up to a distance of 1.9 mm, followed by a more pronounced decline beyond this threshold. After correcting the signal for the roll off, the confocal parameters were estimated.

**FIGURE 4:**
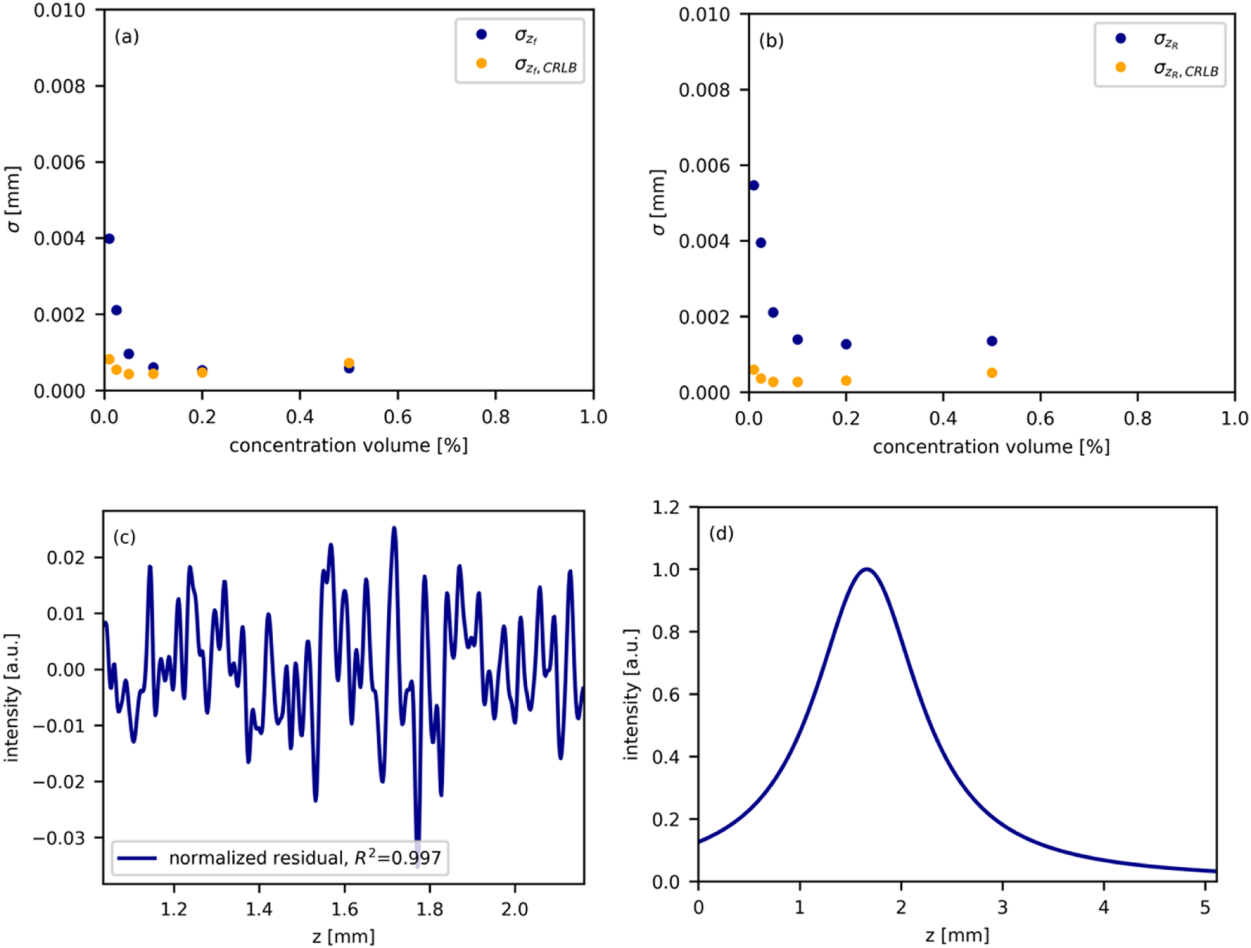
Analysis for deriving confocal parameters. (a, b) display CRLB and standard deviation from the four‐parameter fit (Equation 1) to average A‐scans (*N* = 12 500). A 0.2% concentration volume was chosen for its precision and proximity to theoretical bounds. (c) shows normalized residuals, confirming a stable fit ($R^{2}$ = 0.997). $z_{f}$ is at 0.9250 ± 0.0005 mm with a $z_{R}$ of 0.3516 ± 0.0013 mm. (d) illustrates the resulting normalized point spread function, with peak shift to 1.6 mm due to refractive index correction *n* = 1.34.

Figure 4 illustrates the analysis conducted to derive the confocal parameters. Figure 4a,b display the CRLB and the standard deviation obtained from the four‐parameter fit, Equation (1), respectively. A concentration volume percentage of 0.2% was selected for extracting the confocal parameters due to its demonstration of the highest experimental precision and its proximity to the corresponding numerical CRLB.

Figure 4c showcases the normalized residual of the four‐parameter fit to the data at the 0.2% concentration, indicating a stable goodness of fit, as further confirmed by the high coefficient of determination ($R^{2}$ = 0.997). The extracted focal point position in air ($z_{f}$) is determined to be 0.9250 ± 0.0005 mm, with a Rayleigh length in air ($z_{R}$) of 0.3516 ± 0.0013 mm. Additionally, the resulting plot of the normalized point spread function as a function of optical depth is depicted in Figure 4d. Notably, the shift of the peak to 1.6 mm can be attributed to the correction of the refractive index (*n* = 1.34).

**FIGURE 5:**
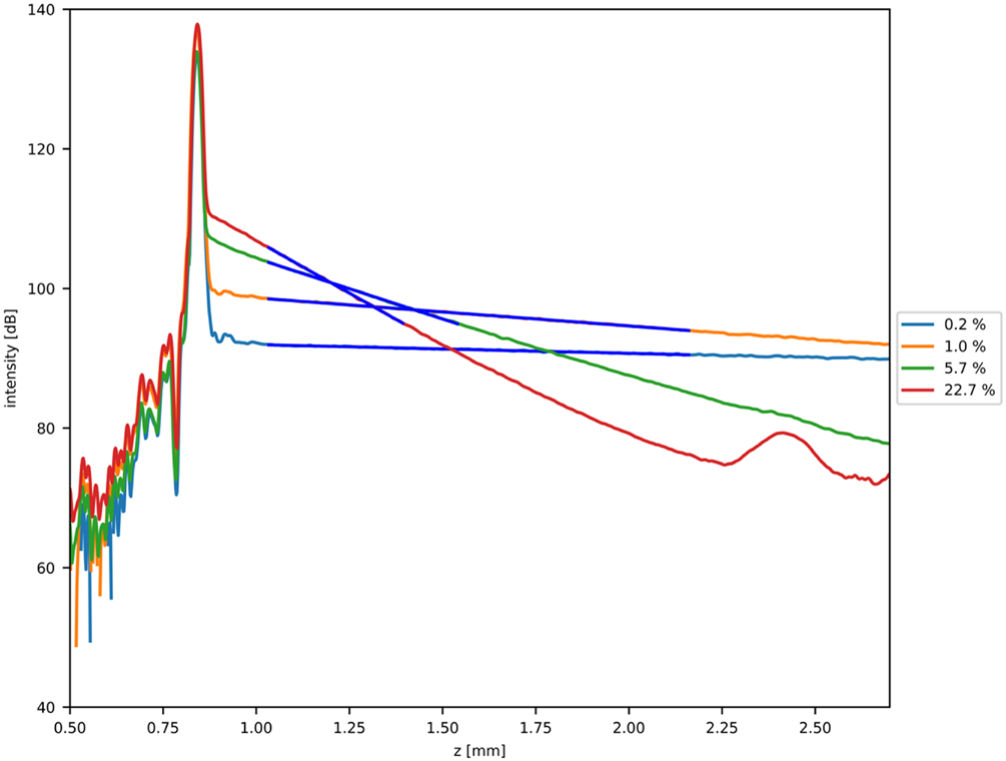
Average A‐scans (*N* = 12 500) after correction for roll‐off, point spread function, and noise offset, shown for a selected range (0.2%, 1%, 5.7%, and 22.7%) of intralipid dilutions to maintain clear visibility. Blue curves represent two‐parameter fits, Equation (3), in dB scale. The corrected A‐scans show expected linear profile post‐correction, transitioning to exponential decay in linear scale. Please note that the width of the fitting range $\left|\mathrm{AFR}\right|$ decreases with concentration to assure a SNR above 20 dB. Residual offset persists post‐noise correction, and at lower concentrations, exponential decay does not fully reach noise floor observed in higher concentrations. System artifacts are visible at depth of 2.4 mm for the highest concentrations.

The average A‐scans (*N* = 12 500), corrected for roll‐off, point spread function, and noise offset, shown for a selected range (0.2%, 1%, 5.7%, and 22.7%) of intralipid dilutions to maintain clear visibility, are depicted in Figure 5. The full range of intralipid dilutions is provided in Appendix A. The blue lines represent the corresponding two‐parameter fit, Equation (3). All graphs are presented in dB scale. As expected (by Equation 3), the A‐scans (in dB) decay linearly in depth, with increasing decay with increasing intralipid concentration.

The fitting ranges vary between 0.36 mm (*M* = 194 pixels) and 1.128 mm (*M* = 600 pixels) to assure a SNR > 20 dB. Despite noise correction, an offset remains visible. Furthermore, for lower concentrations, the exponential decay does not fully reach the noise floor at the same depth position as observed in the higher concentrated samples. At the highest concentration, system artifacts become apparent within the noise floor.

**FIGURE 6:**
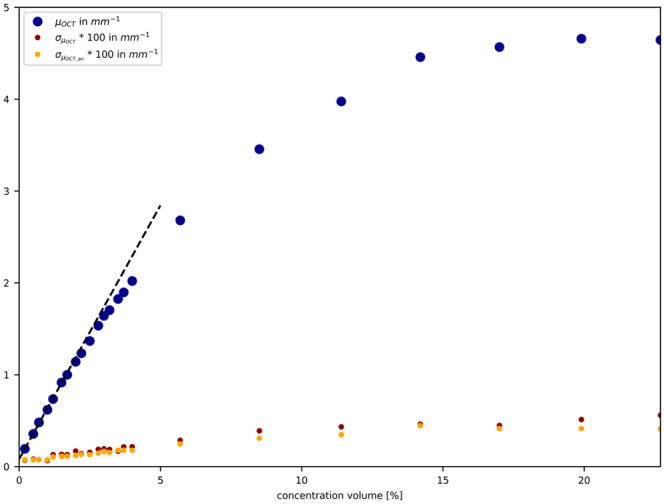
Method 1: Comparison of $\mu_{\mathrm{OCT}}$ (blue dots) and $\sigma_{\mathrm{OCT}}$ (red dots) obtained from the two‐parameter fit, Equation (3) to the *N* = 12 500 average A‐scans. The orange dots represent the analytical CRLB, Equation (6), indicating the theoretical best precision achievable $\sigma_{\mu_{\mathrm{OCT}}}$. The dashed line represents the linear regression of the first five concentration volumes, resulting in an intercept of $\mu_{a}=0.08\;mm^{-1}$.

In Figure 6, we depict the determined $\mu_{\mathrm{OCT}}$ (blue dots) as a function of intralipid concentration using the two‐parameter fit, Equation (3) for the *N* = 12 500 averaged A‐scans. The effect of the dependent scattering is clearly visible by the non‐linear behavior of $\mu_{\mathrm{OCT}}$ with concentration. Furthermore, the standard deviations of the estimated $\mu_{\mathrm{OCT}}$, (red dots), are shown in Figure 6. $\mu_{a}$ is determined by the intercept of a linear regression over the first 5 concentration volumes, which is illustrated as black dashed line. The orange dots represent the analytical CRLB, Equation (6), indicating the theoretical best precision achievable $\sigma_{\mu_{\mathrm{OCT}}}$. For all concentrations, the $\sigma_{\mu_{\mathrm{OCT}}}$ are close to the theoretical predicted CRLB.

**FIGURE 7:**
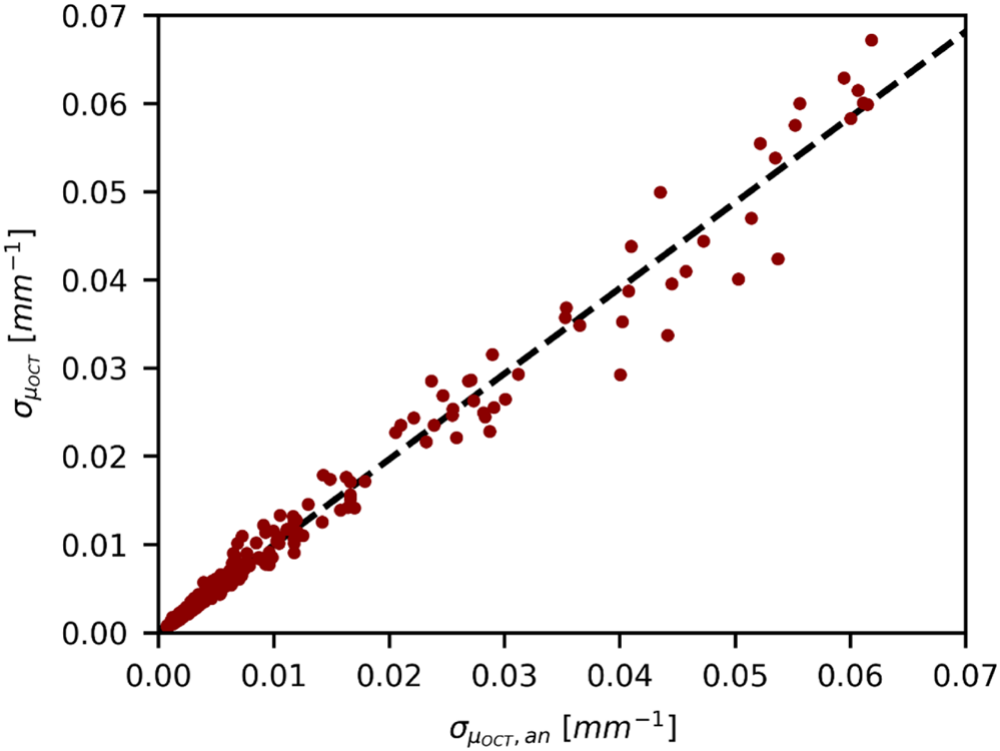
Plot of the measured $\sigma_{\mu_{\mathrm{OCT}}}$ as a function of the expected $\sigma_{\mu_{\mathrm{OCT},\text{an}}}$ based on the CRLB, Equation (6), for all IL concentrations and a range of averages using Method 1. The linear regression, depicted by the dashed black line, shows a slope of 0.9795 ± 0.0008 and an intercept of 0.0002 ± 0.0002 $mm^{-1}$. The goodness of fit, $R^{2}$ is 0.979.

In Figure 7, we plotted the measured $\sigma_{\mu_{\mathrm{OCT}}}$ as a function of the expected $\sigma_{\mu_{\mathrm{OCT}}}$ (based on the CRLB) as obtained for all IL concentrations and for a range of averages. The linear regression, represented by the black dashed line, shows a slope of 0.9795 ± 0.0008 and an intercept of 0.0002 ± 0.0002 $mm^{-1}$. The goodness of fit is, $R^{2}$, is 0.979, indicating an excellent agreement between the experimentally determined standard deviation $\sigma_{\mu_{\mathrm{OCT}}}$ and the analytical CRLB ${\sigma_{\mu }}_{\mathrm{OCT},\text{an}}$.

**FIGURE B1:**
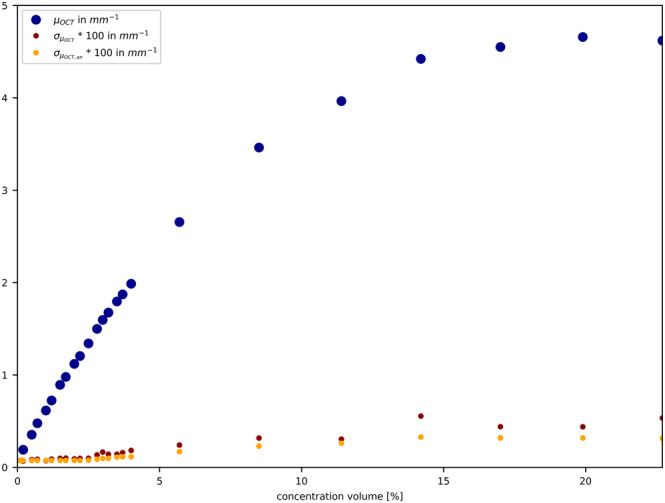
Method 2: Comparison of $\mu_{\mathrm{OCT}}$ (blue dots) and $\sigma_{\mathrm{OCT}}$ (red dots) obtained from the two‐parameter fit, Equation (3). The orange dots represent the theoretical best precision (CRLB). An attenuation value of 0.19 $mm^{-1}$, extracted for the 0.2% intralipid concentration ($\mu_{\mathrm{OCT},0.2}$) from Method 1, has been added as an offset to the results obtained from Method 2.

In Figure B1, provided in Appendix B, we offer further insights into the outcomes when employing Method 2. Despite observing a slight reduction in precision, the results appear to remain consistent with those obtained through Method 1.

The attenuation values exhibit nearly linear behavior in the low concentration regime (< 3 $mm^{-1}$) and converge towards 4.5 $mm^{-1}$ at higher concentrations. The results are equivalent compared to Method 2 (Appendix B). However, in Method 2, an offset of 0.19 mm^−1^ was added to account for absorption $\mu_{a}$ and scattering $\mu_{s}$ ($\mu_{\mathrm{OCT}}=\mu_{a}+\mu_{s}$) for the reference intralipid concentration (0.2%) used to determine the combined roll‐off and point spread function.

**FIGURE 8:**
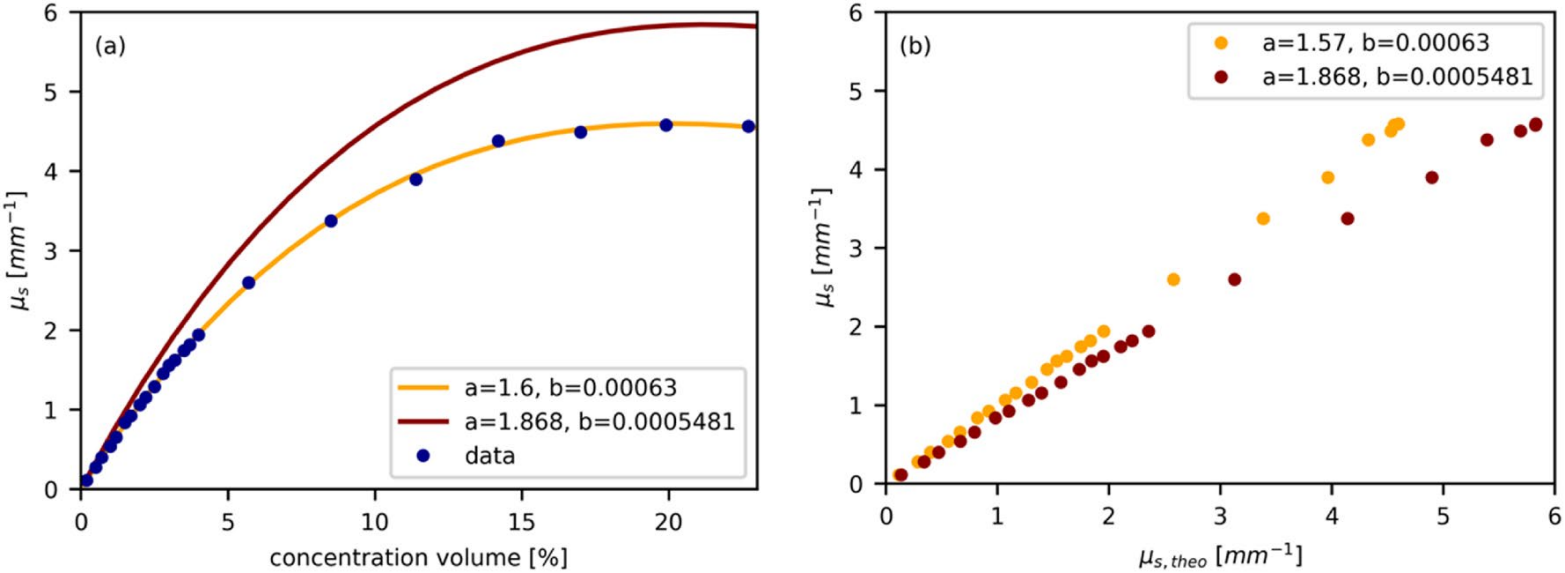
Comparison of experimental values and predictions. (a) Comparison between experimental $\mu_{s}$ (depicted as blue dots), and predicted $\mu_{s}$ (Equation 7) using parameters *a* = 1.868 $nm^{-1}$ and *b* = 0.0005481 $mm^{-1}$ (represented by the red curve), and with adjusted parameters *a* = 1.6 and *b* = 0.00063 (orange line). (b) Direct comparison illustrating the parameter shift, where an overestimation of the theoretical predicted $\mu_{s}$ from Aernouts et al. ([21], red dot), compared to a shift of the parameter values *a* and *b* (orange dots), becomes evident.

Notably, our experimental values (depicted as blue dots) fall below the predicted values based on Equation (7), the model of Aernouts [21], which are illustrated as red line and dots in Figure 8a,b. However, by employing Equation (7) and allowing parameters *a* and *b* to vary freely, the prediction can be aligned with the experimental determined scattering coefficients (orange line), as shown in Figure 8a. Specifically, adjusting parameter *a* to 1.60 (± 0.0074) $nm^{-1}$ and parameter *b* to 0.00063 (± 7.6 × 10^−6^) $mm^{-1}$ shifts the predicted $\mu_{s}$ (depicted as the red line) to closely match our experimental results. The direct comparison before and after shifting of the parameter values is illustrated in Figure 8b, where an overestimation of the predicted $\mu_{s}$ proposed by Aernouts et al. [21] becomes clearly visible.

## 5 Discussion

The presented study explores experimentally the precision in the estimation of the OCT‐attenuation coefficient. Our findings demonstrate the validity of the previously determined CRLB. Furthermore, we describe several key aspects of the measurement processing and analysis process and provide insights into the behavior of the analyzed parameters.

### 5.1 Roll‐Off Data Analysis

The analysis of roll‐off data in Figure 3 reveals a gradual decrease in total roll‐off up to a distance of 1.9 mm, followed by a steeper decline beyond this distance. As we focus solely on correcting the roll‐off accurately rather than determining the roll‐off parameter itself and to ensure accuracy in correcting the entire roll‐off decay, we opted for a 10th‐order polynomial fit. Unlike the model presented in [23], our approach accounts for small fluctuations in the roll‐off, which are, for example, arising due to system and processing artifacts, such as filtering and internal reflections.

### 5.2 Confocal Parameters

The analysis conducted to derive confocal parameters, including the CRLB and standard deviation obtained from the four‐parameter fit, is illustrated in Figure 4. Our selection of a concentrations volume percentage of 0.2% for parameter extraction demonstrates the highest experimental precision and proximity to numerical CRLBs. This high experimental precision is substantiated by the normalized residual as well as the high coefficient of determination ($R^{2}$ = 0.997). We also measured a $z_{R}$ of 0.37 mm using the beam analyzer (Thorlabs BP209‐IR/M), which is in good agreement with our measurement of $z_{R}=0.35\;\mathrm{mm}$. The discrepancy from the beam analyzer can be attributed to the challenge of achieving accurate and precise measurements, as the analyzer is designed for continuous sources while our system is based on a swept source. Consequently, fluctuations in the measured Rayleigh length can occur due to the sweeping nature of the laser source. Notably, it is important to mention that our research did not measure the factor of 2 in front of the Rayleigh length (Equation 2) to account for non‐specular reflection and is therefore conform with [24].

### 5.3 A‐Scan Profiles

**FIGURE C1:**
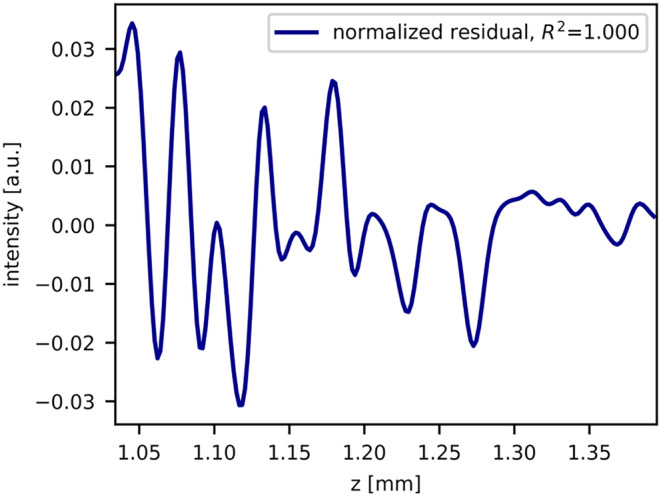
The illustration of residuals for the 22.7% intralipid dilution demonstrates a stable fit (*R* ^2^ = 1.00) using model Equation (3). This result confirms that our assumption of single scattering is adequate.

The A‐scans depicted in Figure 5, after correction for noise offset, roll‐off and point spread function, exhibit a nearly linear profile in dB scale, corresponding to the exponential decay predicted by Equation (3). The relatively flat curve observed for the 0.2% concentration post‐correction aligns with our expectations, suggesting a minimal attenuation coefficient. However, as the concentration increases, a more rapid decline in the corrected A‐scans with concentration is anticipated due to the increased OCT attenuation per concentration. Despite the noise‐offset correction, there remains a small offset visible in Figure 5. In theory, this offset has no impact/influence on the measured parameters and corresponding standard deviations. Furthermore, our experimentally determined attenuation coefficients show deviations from the predictions [21]. As discussed in [25], these deviations may be attributed to multiple scattering phenomena. Specifically, [25] suggests that the measured intralipid data falls below theoretical expectations predicated on single scattering assumptions, a trend consistent with our observations. Furthermore, the near‐linear behavior exhibited by attenuation values in the lower concentration regimes aligns with previous research [25]. However, using the fitting routine assumes that our sample is homogeneous within the region of interest. The residuals of the fit, thoroughly analyzed in Appendix C (Figure C1), show a strong alignment between the fitted model, Equation (3), and the experimental data. This outcome supports our assumption that single scattering events are sufficient, countering the argument for the presence of multiple scattering. Additionally, any deviations in accuracy may suggest that the sample is not perfectly homogeneous. Bodenschatz et al. [26] provided evidence of a time‐dependent thin scattering layer forming on the intralipid surface. This layer should be accounted for, such as with a two‐layer model, when using intralipid as a reference phantom.

Our experimentally derived standard deviation of $\mu_{\mathrm{OCT}}$ closely matches our analytical prediction, Equation (6), indicating agreement between experimental and theoretical results. Assuming negligible influence of multiple scattering in, especially in lower regimes, and single scattering predominance, we experimentally validated our previously published analytical CRLB, Equation (6). In Figure 7, the one‐to‐one correlation between the expected and measured $\sigma_{\mu_{\mathrm{OCT}}}$ clearly shows experimental confirmation of the CRLB analysis.

However, challenges in accuracy and precision emerged in using the depth‐resolved technique [27]. Notably, our DRE results were sensitive to the estimation of the $\mu_{\mathrm{OCT}}$ at the fitting range's end. While the two‐parameter fitting procedure informed these estimations, reliable determination of in‐depth attenuation coefficients proved challenging.

Furthermore, our findings based on Method 1, where we determined the confocal parameters and roll‐off decay individually, demonstrate a rigorous approach to obtaining precise measurements. However, we also identified a more practical method using a 0.2% intralipid concentration for correction across individual concentrations. While this simplified method is straightforward, our research, detailed in Appendix B, demonstrates that it achieves attenuation coefficient values with precision and accuracy comparable to Method 1. It is important to note, that an offset due to absorption and scattering at this concentration must be added to the attenuation value in Method 2. Furthermore, after the calibration Method 2, tissue surface and/or the focus positions have to be in the same positions relative to zero‐delay throughout measurements. Consequently, Method 1 may have a broader range of applications compared to Method 1.

### 5.4 Application and Clinical Relevance

Certain research inquiries necessitate precise measurements to yield conclusive answers, particularly within the medical domain where even slight variations, such as alterations in human hydration levels, or the level of oxygen saturation in, for example, blood [28, 29], significantly impact health outcomes. In this study, we illustrate the feasibility of achieving requisite precision levels using OCT. Through experimental validation, we have established that our precision measurements closely approximate the CRLB, as depicted by the one‐to‐one correlation between the expected and measured $\sigma_{\mu_{\mathrm{OCT}}}$ in Figure 7. Our analytical investigations further reveal that this lower bound can be mitigated by increasing the number of averages, expanding the number of independent sampling points, and widening the region of interest. Consequently, the latter parameter must be adequately sized during attenuation coefficient determination via fitting to ensure sufficient information inclusion.

However, this requirement for region width underscores the need of sample homogeneity throughout the region of interest. In biological tissue investigations, achieving such homogeneity poses challenges, especially when striving for extremely high precision.

A practical and challenging example of the importance of precision in attenuation coefficient measurements lies in the assessment of the hydration state of tissue. In order to monitor the hydration status of people, for example, during exercise in warm environments, or elderly at home, as well as patients in hospitals, a method to determine the water content of tissue with a precision of at least 2% is needed [30, 31, 32, 33]. Previously, we formulated a theoretical framework for assessing tissue hydration using parametric imaging through a triple‐wavelength OCT system. Our findings indicated that to achieve a clinically meaningful precision of 2% in the water fraction, the attenuation coefficient must be determined with a precision of 0.01 $mm^{-1}$ [34]. One significant challenge in translating our findings to clinical settings is the variability of human tissue. Our study focused on homogeneous samples to validate precision experimentally. We demonstrated that the desired precision is achievable in sufficiently homogeneous regions, determined by the number of A‐scans used for lateral averaging, *N*, which improves precision by a factor of $1/\sqrt{N}$. The appropriate value of *N* can be selected based on the research objectives, using the analytical equations [15, 19]. For example, a data fitting range of 0.5 mm, with an independent sampling size of 10 μm (including the starting point), results in 51 independent sample points. Using the analytical CRLB equation for the fitting method [15], 2565 A‐scans are required for lateral averaging. With a spot size of 20 μm on the sample surface, the scanning area must cover at least a sample surface area of approximately 1 mm by 1 mm in order to extract sufficiently precise attenuation coefficients for determining the water fraction. Further research is needed to identify representative tissue areas that are large and homogeneous enough to ensure reliable measurements.

Variability in location within biological samples presents an additional challenge. Since our approach relies on localized measurements, accurately determining an individual's overall hydration status requires identifying a body region that reliably reflects total body water content, an especially difficult task given the uneven distribution of water across various body parts and organs. Moreover, for effective clinical use, the feedback loop for monitoring hydration must be rapid enough to provide timely guidance, adding further complexity to the application of our methods. Identifying a measurement location that accurately represents the body's overall water content while being sufficiently homogeneous is a challenging yet feasible task. Instead of measuring the exact amount of water, an alternative approach is to obtain precise and accurate readings relative to a healthy reference. Achieving this will likely require further interdisciplinary research, and the potential applications extend beyond the medical field.

In addition to averaging, other techniques such as angular compounding [35, 36], where speckle reduction is achieved by averaging multiple images acquired at different angles, could be implemented to reduce speckle noise. This, in turn, would decrease the amount of tissue area required for precise measurements.

Another challenge lies in the fitting range. Our demonstration primarily relied on OCT attenuation determined through the curve‐fitting method, which requires a certain sample layer length to fit data over a specified region of interest. A longer fitting range provides more data points, incorporating additional information and thereby enhancing precision. However, applications focusing on thin‐layered samples, such as ophthalmology, may face difficulties in precisely extracting the attenuation coefficient using this approach. In such cases, the Depth‐Resolved Estimation (DRE) method may be more suitable, as it determines an attenuation value at each pixel depth rather than averaging over a region of interest. However, to obtain a precise value of the attenuation coefficient averaging over a large number of pixels in a layer is still needed. Achieving a high precision in a smaller region of interest will require an increased number of averages, which in turn would also increase the required tissue area. Furthermore, due to potential bias near the end of the imaging range, challenges arise in accurately determining OCT attenuation [19].

Nevertheless, in instances where sample homogeneity is sufficiently met, our research demonstrates that attenuation coefficients can be determined with the requisite precision, approaching the analytical CRLB. Additionally, in our study, we opted for a relatively high number of A‐scans for averaging. However, the number of averages may need to be adjusted depending on the specific measurement requirements and characteristics of the sample under investigation. In Appendix D, we demonstrate that highly precise OCT measurements can still be achieved even when using a reduced number of A‐scans for averaging.

Therefore, our experimental findings validate the applicability of the analytical solution in defining measurement requirements and contribute to improving the precision of experimentally derived attenuation coefficients. Additionally, we offer a systematic framework for achieving high precision measurements, aiming to enhance methodological standards in the field.

## 6 Conclusion

The primary objective of this study was to experimentally validate the analytical CRLB and the theoretical framework derived in [15]. Our experimental findings confirmed the validity of the analytical CRLB, demonstrating unprecedented precision in OCT‐attenuation measurements, with a standard deviation of less than 0.01 mm^−1^ for intralipid samples. This achievement, attained through rigorous data post‐processing and insights from prior research, serves as a proof of concept in a homogeneous medium, supporting its applicability for ensuring reliable clinical measurements. Furthermore, we introduce a systematic framework to consistently achieve such high precision in measurements.

## Conflicts of Interest

The authors declare no conflicts of interest.

## Figures and Tables

**FIGURE A1:**
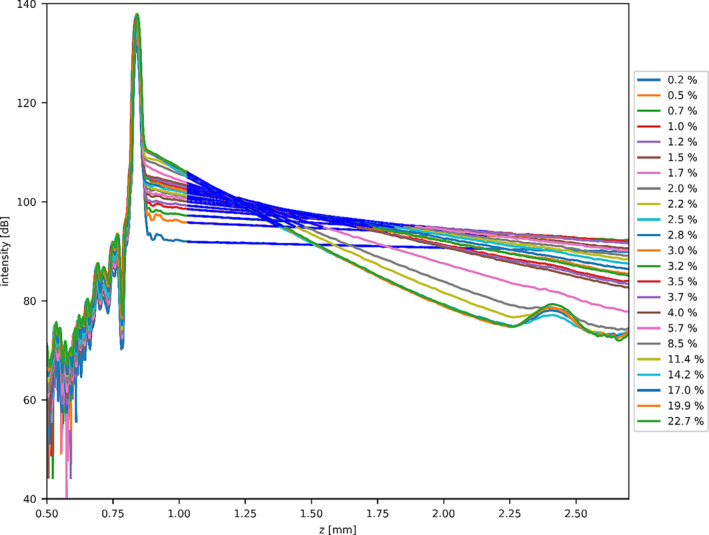
Averaged A‐scans (*N* = 12 500) following corrections for roll‐off, point spread function, and noise offset. The blue curves correspond to two‐parameter fits based on Equation (3), presented on a dB scale. The corrected A‐scans exhibit the anticipated linear decay profile after correction, transitioning to an exponential decay when plotted on a linear scale. It should be noted that the width of the fitting range (∣AFR∣) decreases with increasing concentration to maintain an SNR above 20 dB. Despite noise correction, a residual offset remains. At lower concentrations, the exponential decay does not fully reach the noise floor observed in higher concentrations. System artifacts are apparent at a depth of 2.4 mm for the highest intralipid concentrations.

**FIGURE B2:**
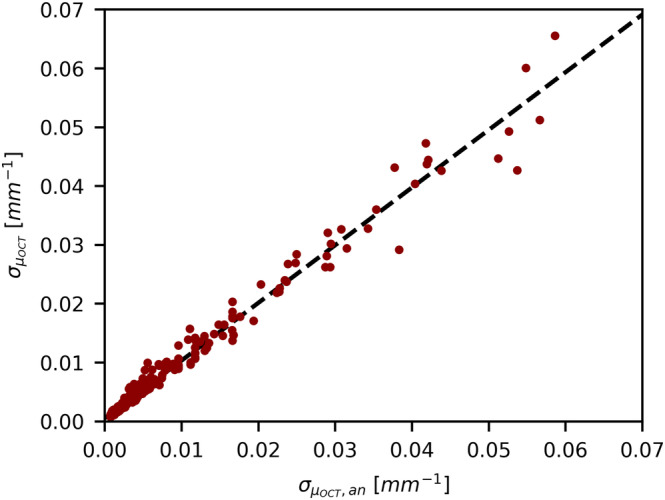
Plot of the measured $\sigma_{\mu_{\mathrm{OCT}}}$ as a function of the expected $\sigma_{\mu_{\mathrm{OCT},\text{an}}}$ based on the CRLB, Equation (6), for all IL concentrations and a range of averages, using Method 2. The linear regression, depicted by the dashed black line, shows a slope of 0.9802 ± 0.0070 and an intercept of 0.0005 ± 0.0001 mm. The goodness of fit, $R^{2}$ is 0.98.

**FIGURE D1:**
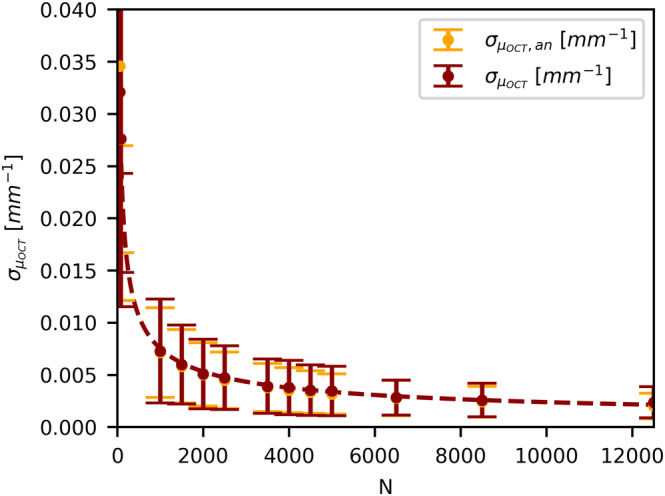
Illustration of the experimentally determined standard deviation (red dots) extracted from the two‐parameter fit, Equation (3), and the analytical lower bound (orange dots), Equation (6), as a function of the number of A‐scans taken for averaging, using Method 1. The error bars depict the range of variations in both analytical and experimental precision across different concentrations.

**FIGURE D2:**
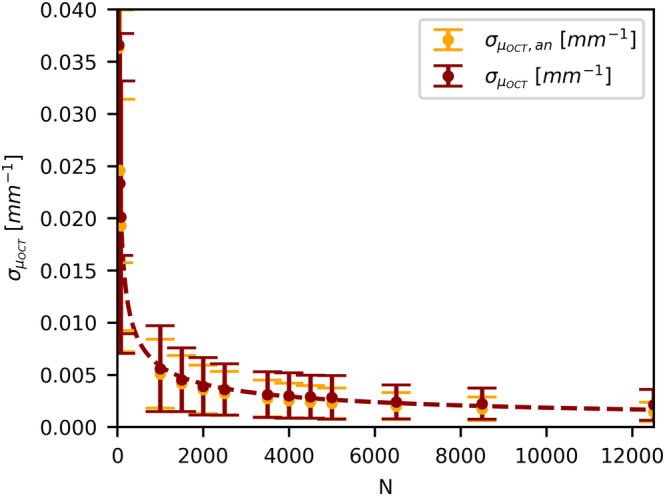
Comparison of the experimentally determined standard deviation (red dots), obtained from the two‐parameter fit (Equation 3), and the analytical CRLB (orange dots), calculated using Equation (6). Both are plotted as a function of the number of A‐scans used for averaging, based on Method 2. The error bars depict the range of variations in both analytical and experimental precision across different concentrations.

## Data Availability

The data that support the findings of this study are available from the corresponding author upon reasonable request.

## Appendix A

Figure A1 depicts the averaged A‐scans (*N* = 12 500) after applying corrections for roll‐off, point spread function, and noise offset. The data includes the complete set of intralipid dilutions. The blue lines represent the two‐parameter fit described by Equation (3). All graphs are presented on a dB scale. As predicted by Equation (3), the A‐scans exhibit a linear decay (in dB) with depth, and the rate of decay increases with higher intralipid concentrations.

The fitting range spans from 0.36 mm (*M* = 194 pixels) to 1.128 mm (*M* = 600 pixels) to ensure a signal‐to‐noise ratio (SNR) above 20 dB. Despite the noise correction, a residual offset remains visible. At lower concentrations, the exponential decay does not fully reach the noise floor at the same depth as observed for higher concentrations. At the highest concentration, system artifacts become evident, manifesting within the noise floor.

## Appendix B

In Figure B1, we offer a comparison between the values of $\mu_{\mathrm{OCT}}$ (depicted as blue dots) and their associated standard deviations (depicted as red dots), obtained from the two‐parameter fit utilizing Equation (3) and Method 2. Additionally, the orange dots illustrate the analytical CRLB, delineated by Equation (6), providing a benchmark for the theoretical best achievable precision $\left(\sigma_{\mu_{\mathrm{OCT}}}\right)$.

It's noteworthy that all concentrations closely approach this theoretical prediction, maintaining consistent alignment with the analytical CRLB. Despite a marginal reduction in precision, the outcomes from Method 2 exhibit a remarkable consistency with those obtained through Method 1. As with Method 1, the validation of the analytical CRLB, using Method 2, is illustrated in Figure B2. The measured $\sigma_{\mu_{\mathrm{OCT}}}$ is plotted against the expected $\sigma_{\mu_{\mathrm{OCT}},\text{an}}$, (derived from the CRLB) for various intralipid concentrations and numbers of averages. The black dashed line represents the linear regression, with a slope of 0.9802 ± 0.0.0070 and an intercept of 0.0005 ± 0.0001 mm^−1^. The goodness of fit, $R^{2}$, indicates 0.98, demonstrating strong agreement between the experimentally obtained standard deviation $\sigma_{\mu_{\mathrm{OCT}}}$ and the analytical CRLB $\sigma_{\mu_{\mathrm{OCT},\text{an}}}$.

## Appendix C

Our model, as described by Equation (3), is based on the assumption of single scattering. While multiple scattering could potentially occur, particularly at higher concentrations, it would likely manifest as an additional exponential decay with a distinct decay rate. However, our residuals exhibit excellent agreement, $R^{2}=1.000$, with the single scattering model, providing no indication that any deviation in the accuracy of the attenuation coefficient is due to multiple scattering events.

## Appendix D

The relationship between precision and the number of A‐scans averaged, *N*, is well‐established, with precision decreasing proportionally to the square root of *N*. This dependency is visually demonstrated in Figure D1 (Method 1) and Figure D2 (Method 2), where we compare the standard deviation of the two‐parameter fit, Equation (3) (depicted as red dots) with the analytical CRLB, Equation (6) (depicted as orange dots).

To compute the standard deviation, we calculate the mean standard deviation across all individual standard deviations of the intralipid concentrations. The error bars represent the range within which the analytical and experimental precision vary among the different concentrations. Hence, *N* is strongly dependent on the sample itself, as it dictates the amount of data, or information, that can be incorporated into the analysis through the width of the AFR. This suggests the potential for achieving slightly greater experimental precision, even with a relatively low number of averages. These findings underscore the capability of OCT to facilitate highly precise non‐invasive measurements, a quality of significant relevance in clinical research.
